# Supplementary material for: Generic wound signals initiate regeneration in missing-tissue contexts
Source: Nat Commun. 2017 Dec 22;8:2282. doi: 10.1038/s41467-017-02338-x (PMC5741630; doi:10.1038/s41467-017-02338-x)
Supplement: Supplementary file 1 — Supplementary Information [file 41467_2017_2338_MOESM1_ESM.pdf]

Supplementary Figures

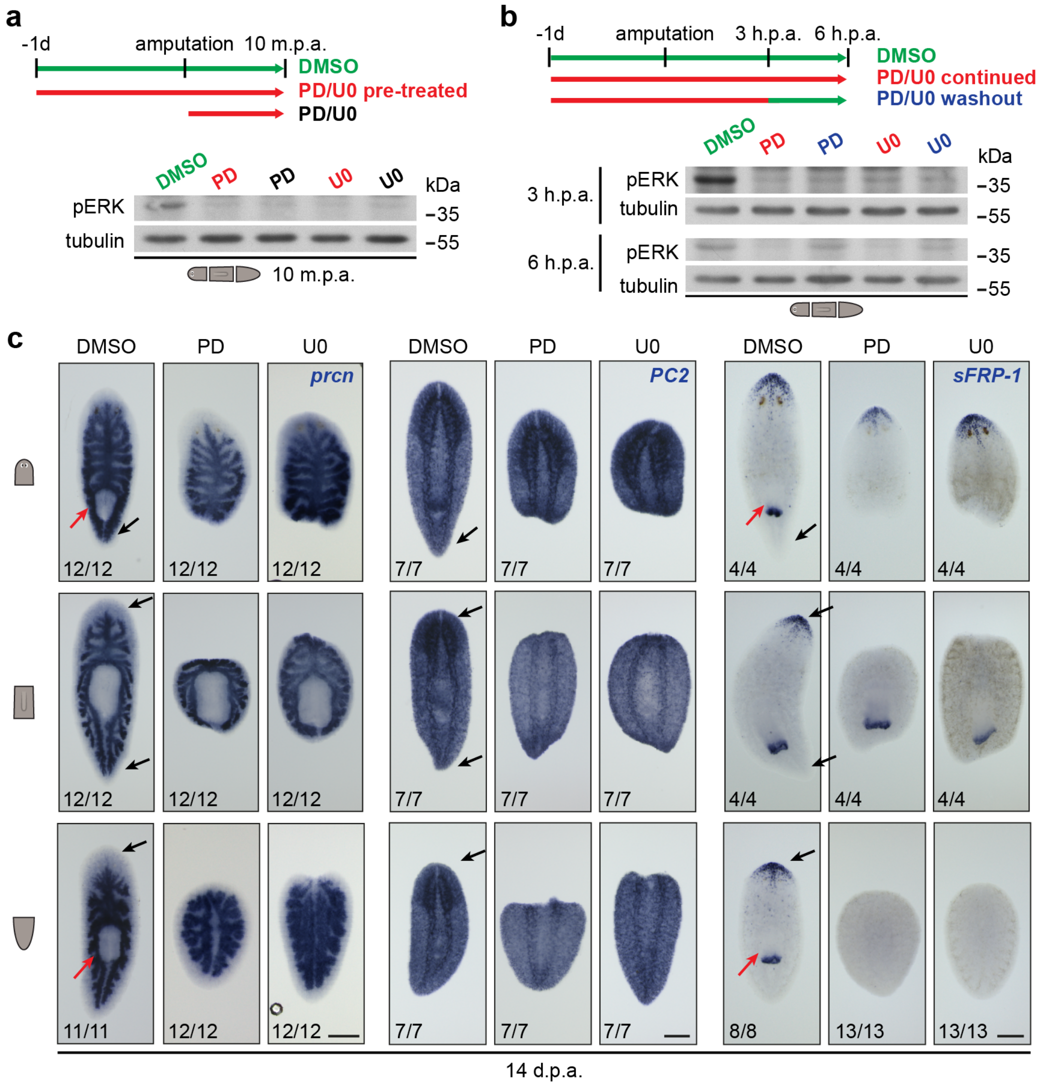

Supplementary Figure 1. MEK inhibitors reversibly inhibit ERK phosphorylation and prevent tissue regeneration

a-b Effects of MEK inhibitors on pERK levels were effective within 10 minutes (**a**) and reversible within 3 hours (**b**). **c** Treatment with PD and U0 inhibited regeneration of new tissues in the blastema (black arrows) as well as remodeling of existing tissues (red arrows). *prcn* (*porcupine*) gut marker, *PC2* (*prohormone convertase 2*) pan-neuronal maker, *sFRP-1* (*secreted frizzled-related protein-1*) anterior marker, m.p.a minutes post-amputation, h.p.a. hours post-amputation, d.p.a. days post-amputation, scale bars: 200 µm

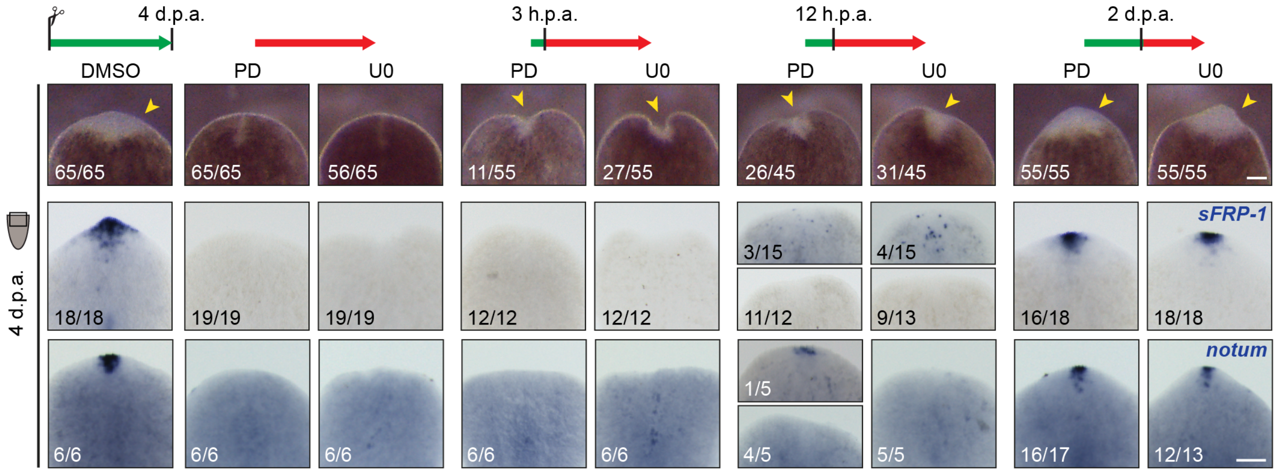

Supplementary Figure 2. ERK activation is required within 12 hours of amputation

Delaying drug treatment start times resulted in partial rescue of blastema formation and anterior marker expression. Bars indicate time windows of drug (red) and DMSO treatment (green); yellow arrowheads indicate the blastemas, *sFRP-1* (*secreted frizzled-related protein-1*) and *notum* are anterior markers; h.p.a. hours post-amputation, d.p.a. days post-amputation,scale bars: 100 µm

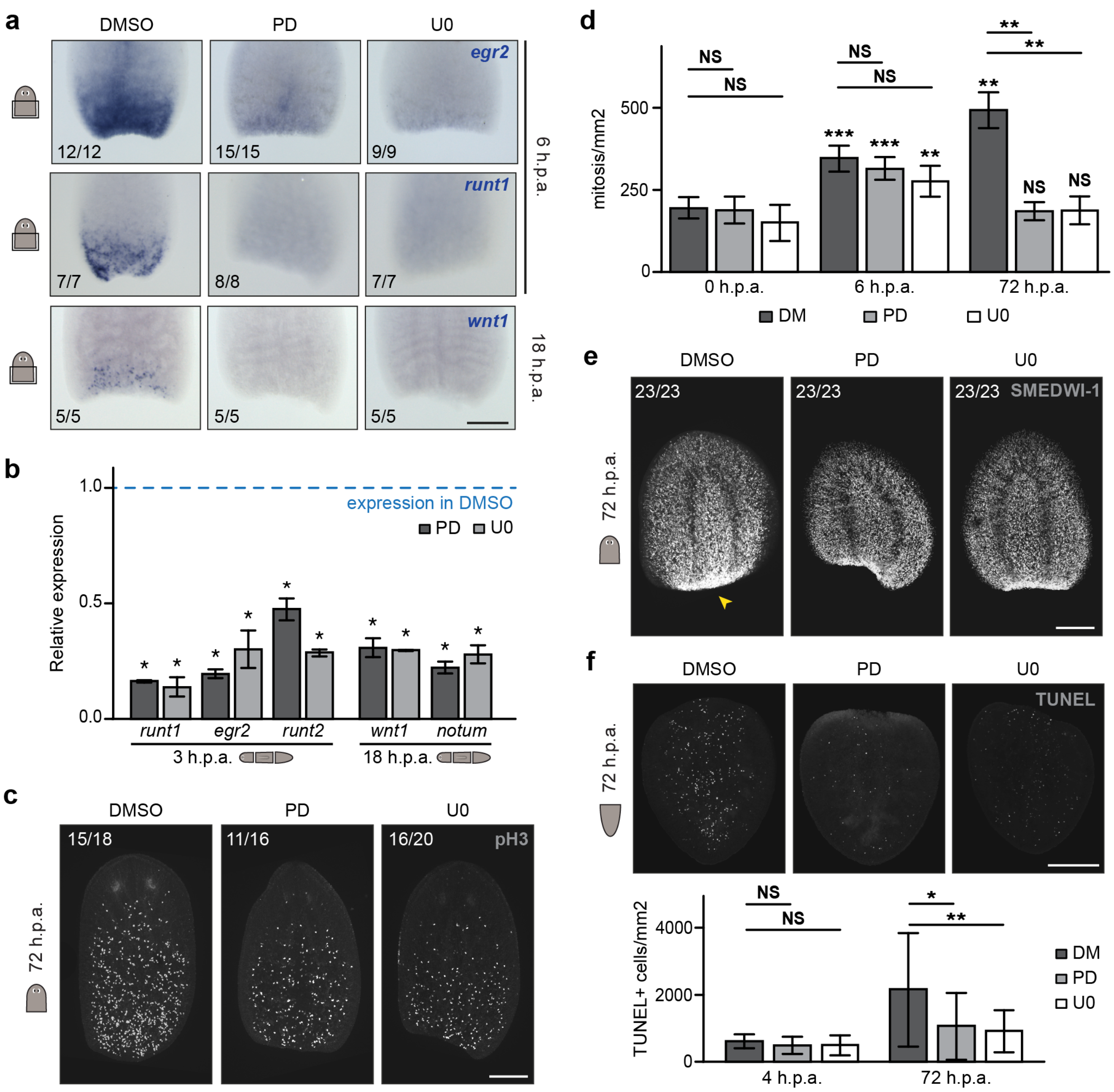

Supplementary Figure 3. ERK signaling is essential for the activation of early regenerative responses

a-b Treatment with PD and U0 prevented expression of wound-induced genes at 3–18 hours post-amputation (h.p.a.). Bars in graphs represent mean of three biological replicates ± standard deviation (s.d.), normalized to DMSO controls (blue line); two-sided t-test, * = p<0.05. **c** Inhibition of ERK activity severely inhibited regeneration-associated proliferation (pH3, phospho-Histone H3). **d** In amputated tail fragments, generic wound-induced proliferative responses (6 h.p.a.) were not significantly affected by inhibition of ERK signaling, while regeneration-associated proliferation (72 h.p.a.) was abolished. Bars in graphs represent mean ± s.d.; two-sided t-test (compared to corresponding 0 h.p.a. samples, unless otherwise indicated), * = p<0.05, ** = p<0.01, NS is not

significant. **e** Accumulation of stem cells and progeny (SMEDWI-1) at the amputation site (arrowhead) was severely affected in absence of active ERK signaling. **f** Late apoptotic response characteristic of tissue remodeling during regeneration, as detected by TUNEL staining, was also strongly reduced. Scale bars: 200 μm

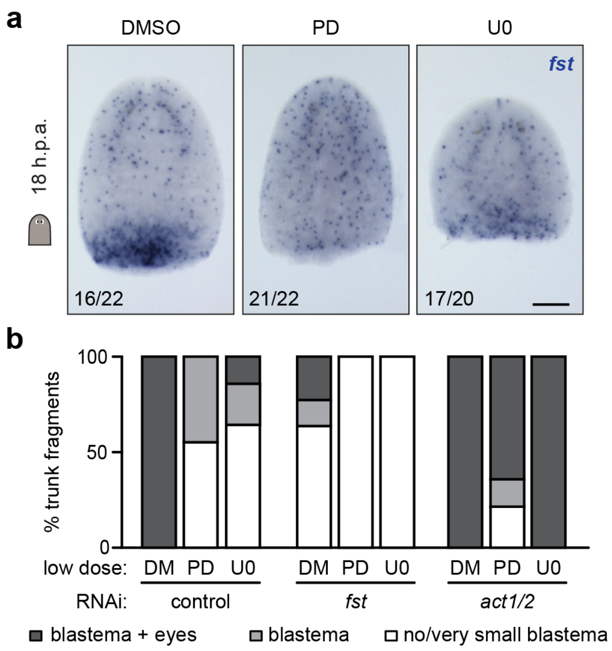

Supplementary Figure 4. ERK signaling may impact regeneration through Follistatin-mediated Activin inhibition

a Wound-induced expression of *fst (follistatin)*, which encodes an Activin inhibitor essential for regeneration initiation in planarians[2], is dependent on ERK activity. **b** Knockdown of *fst* enhanced the regeneration defects caused by low dose PD (1:3 dilution) or U0 (1:2.5 dilution), while double knockdown of *act-1(activin-1)* and *act-2 (activin-2)* partially rescued these defects. n=14-33; scale bar: 200 μm

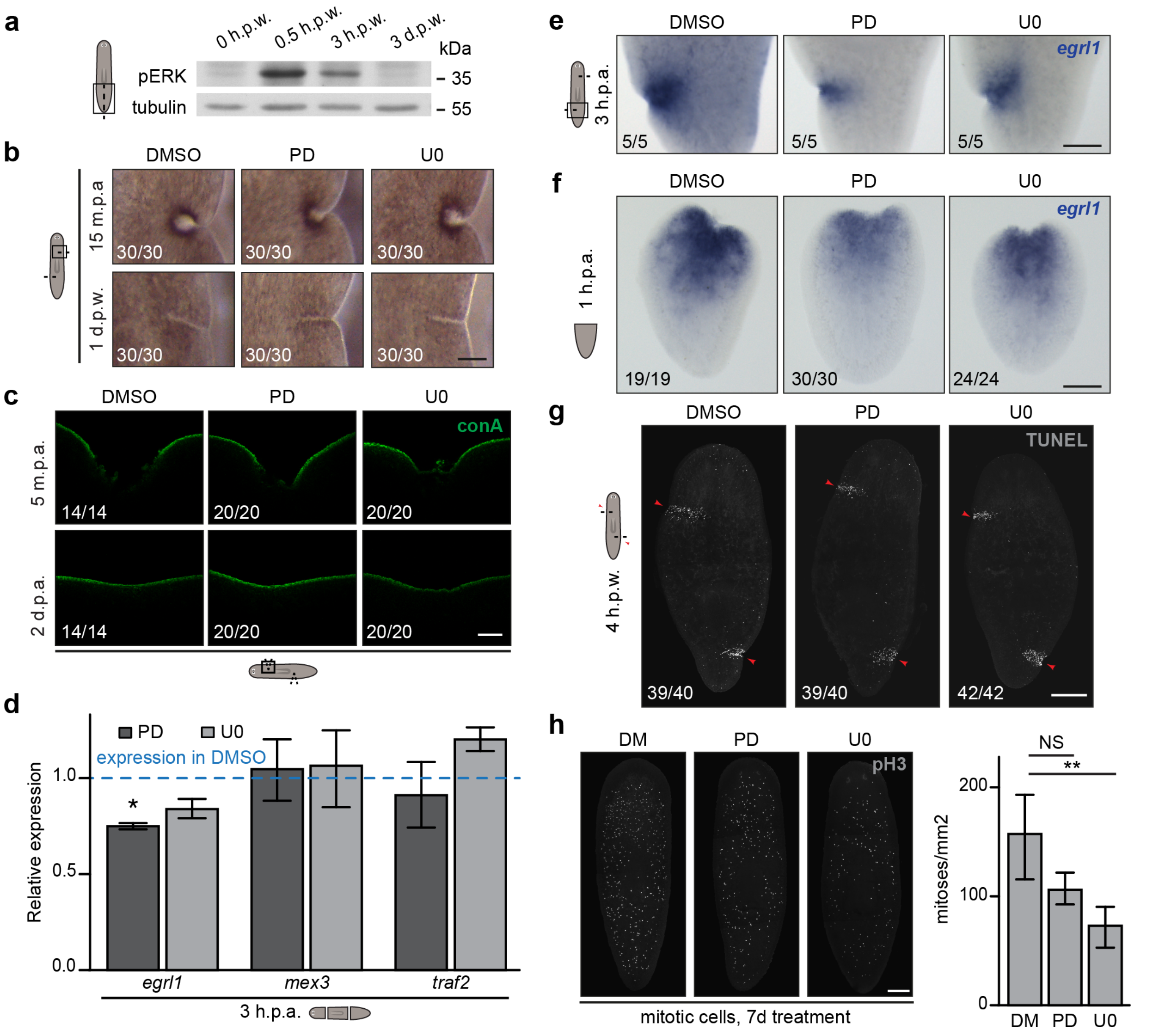

Supplementary Figure 5. Inhibition of ERK signaling allows for wound healing as well as other generic responses to injury

a ERK was activated after H-wound injuries that do not involve tissue loss. **b-c** Wounds heal in the absence of ERK signaling (ConA, Concanavalin-A, epithelial marker [3]). **d-f** ERK inhibition did not prevent the induction of the stress-response genes *egrl1, mex3* and *traf2*[4] after R-wounds (**d, f**), nor after H-wounds (**e**). Note that expression of *egrl1* in drug-treated animals appeared less spread at 3 hours post-injury. **g** Inhibition of ERK activity also did not affect the activation of the early generic wound-induced apoptotic response. **h** Proliferation levels were slightly affected in uninjured animals. Bars in graphs represent mean of three biological replicates ± s.d., normalized to DMSO controls (blue line); two-sided t-test, * = p<0.05, ** = p<0.01, NS is not significant. Numbers refer to injured areas (two per animal); m.p.w. minutes post-wounding, h.p.w. hours post-wounding, d.p.w. days post-wounding; scale bars: **b**, **e-h** 200 µm, **c** 50 µm

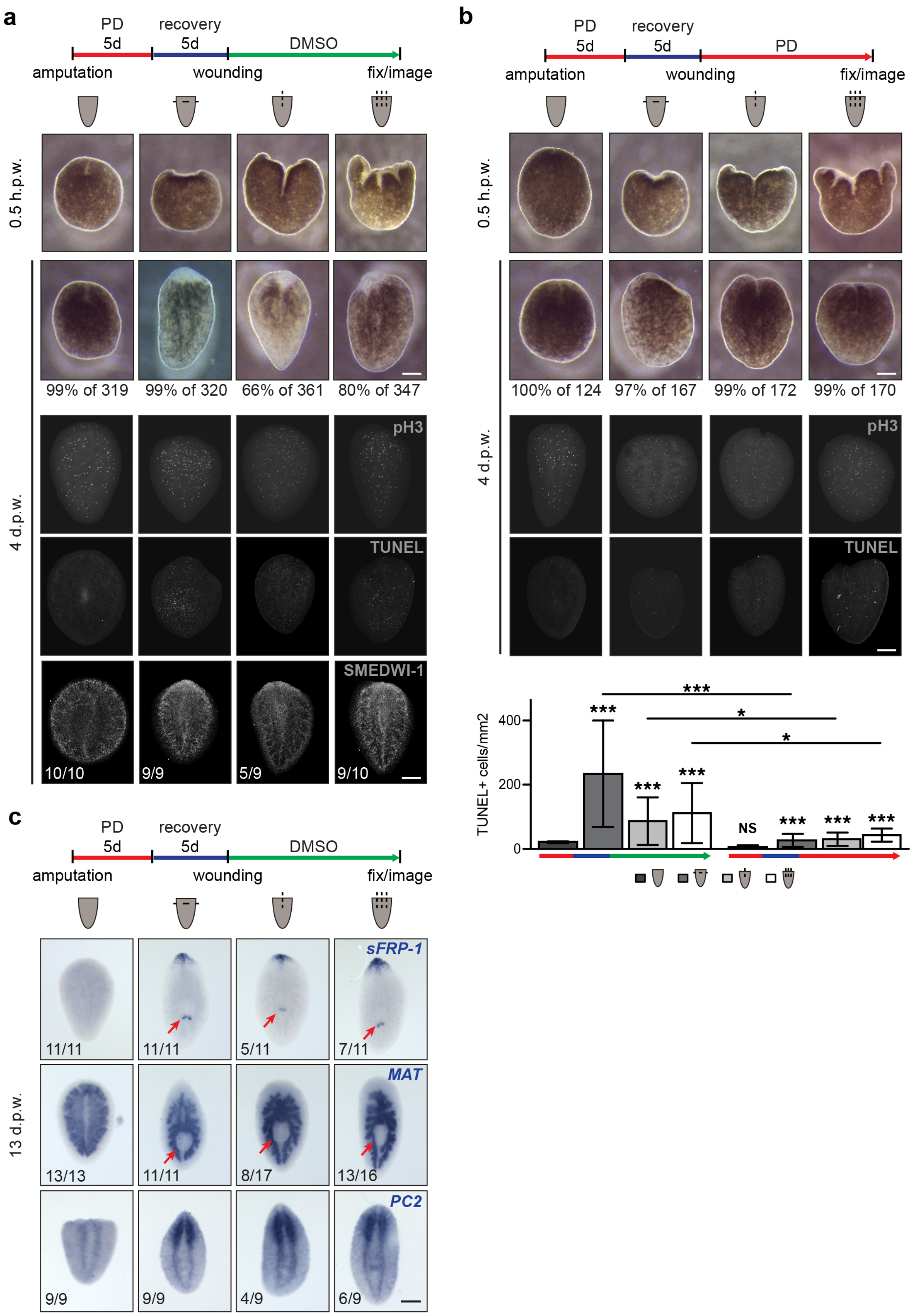

Supplementary Figure 6. Both H- and R-wounds induce similar regenerative responses in rescued dormant tails

a-b Experimental scheme: red indicates PD treatment, blue DMSO treatment during recovery period

before re-injury, and green DMSO treatment after re-injury. Dormant tails rescued by R-wounds and H-wounds induced similar early regeneration-associated responses (**a**) that were blocked by treatment with PD (**b**). **c** This was followed by similar morphological recovery. SMEDWI-1 stem cell and progeny marker; anti-pH3 (phospho-Histone H3) labels mitotic cells; *sFRP-1* (*secreted frizzled-related protein-1*) anterior marker; *MAT* (*methionine adenosyltransferase*) gut marker; *PC2* (*prohormone convertase 2*) pan-neuronal maker; TUNEL assay labels apoptotic cells (proxy for tissue remodeling); red arrows label pharynges. Bars in graphs represent mean ± s.d.; two-sided t-test (compared with uninjured PD-washed-out animals (first bar, dark grey; corresponding to first column of panel a), unless otherwise indicated), * = $p<0.05$, ** = $p<0.01$, *** = $p<0.001$, NS is not significant; scale bars: 200 μm

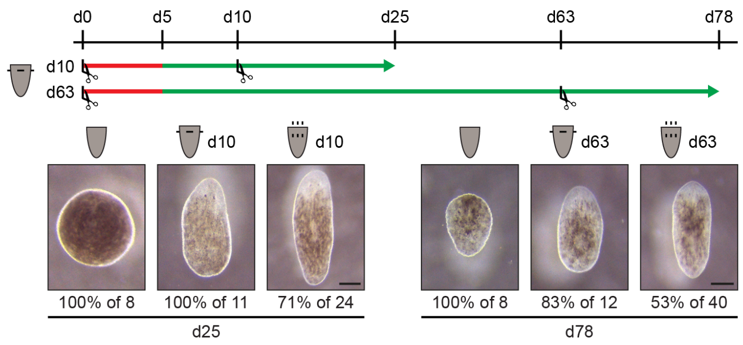

Supplementary Figure 7. New wound signals can initiate regeneration in dormant tails even two months after drug removal

At 5 or 58 days after removal of PD, dormant tails were rescued by both R-wounds and H-wounds. Red color in the scheme indicates PD treatment. Scale bars: 200 μm

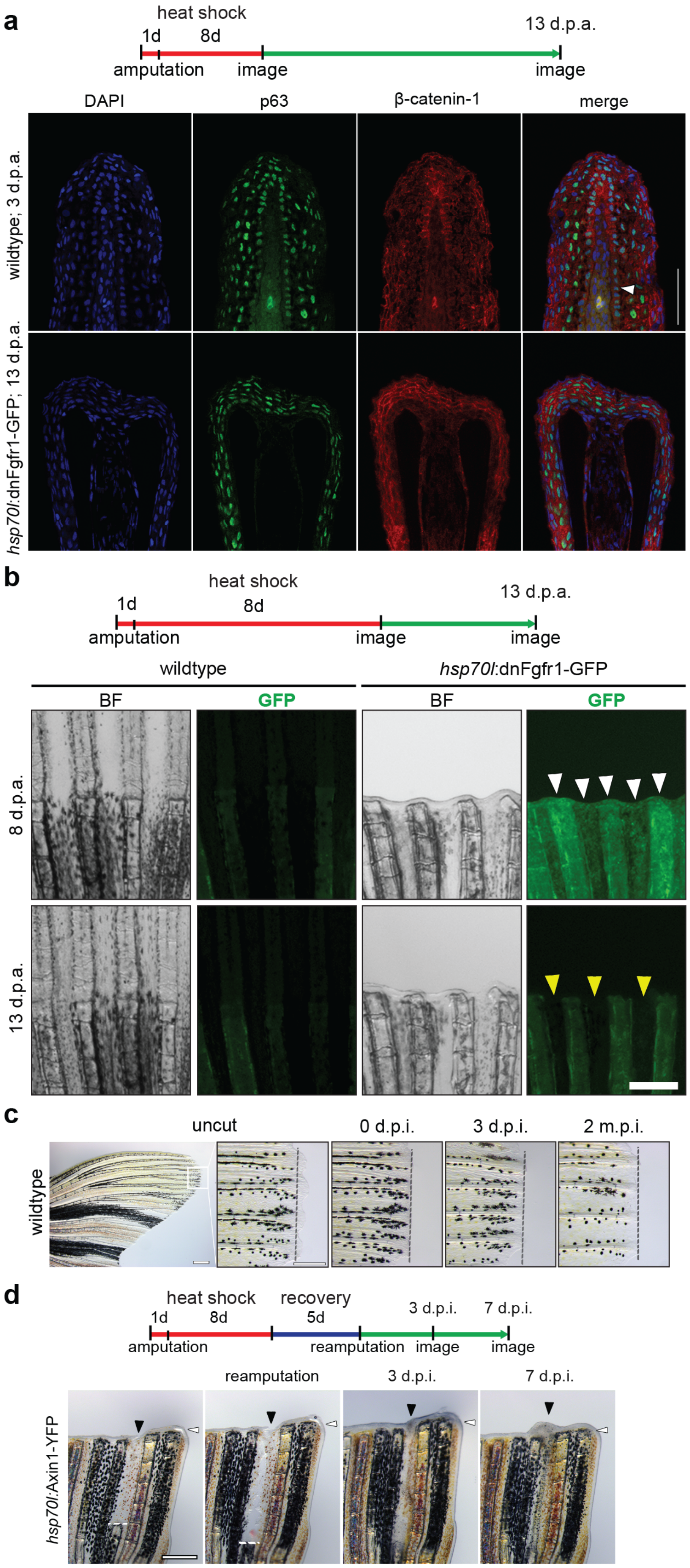
a
heat shock
1d
8d
13 d.p.a.
amputation
image
image
DAPI
p63
β-catenin-1
merge
wildtype; 3 d.p.a.
hsp70l:dnFgfr1-GFP; 13 d.p.a.
b
heat shock
1d
8d
13 d.p.a.
amputation
image
image
wildtype
hsp70l:dnFgfr1-GFP
BF
GFP
BF
GFP
8 d.p.a.
13 d.p.a.
c
uncut
0 d.p.i.
3 d.p.i.
2 m.p.i.
wildtype
d
heat shock
recovery
1d
8d
5d
3 d.p.i.
7 d.p.i.
amputation
reamputation
image
image
reamputation
3 d.p.i.
7 d.p.i.
hsp70l:Axin1-YFP

Supplementary Figure 8. Regenerative responses but not formation of a wound epidermis are blocked in dormant fins

a Longitudinal cryosections stained for the epidermal marker p63 and the cell surface marker β-catenin reveal that formation of a multi-layered wound epidermis did occur in *hsp70l*:dnFgfr1-GFP transgenic fish subjected to the indicated heat-shock regime. However, the basal layer of the epidermis, which is a hallmark of the specialized wound epidermis forming in regenerating fins, is only evident in wild-type fins at 3 d.p.a. (white arrowhead), but did not form in the FGF signaling inhibited fins. **b** Heat shocks applied 4x daily resulted in robust expression of dnFgfr1-GFP in *hsp70l*:dnFgfr1-GFP fish in ray and inter-ray tissue by 8 d.p.a. (white arrowheads). After a 5-day recovery period, GFP expression was no longer detected in inter-ray tissue (yellow arrowheads) and fluorescence in rays had dropped to background levels observed in wild-type fish. BF, bright-field image. **c** Surgical removal of the skin at the distal tip of non-amputated fins did not result in ray growth, even when followed to 2 months post injury (m.p.i.). Dashed line indicates the injury plane. n=17 fish, 67 rays. **d** Re-amputation of individual rays induced regenerative growth in recessed rays (8 of 8 rays) of dormant *hsp70l*:Axin1-YFP transgenic fins (black arrowheads), white arrowheads indicate the primary amputation plane. n = 8 fish. Scale bars: a, 100 μm; c,d, 500 μm

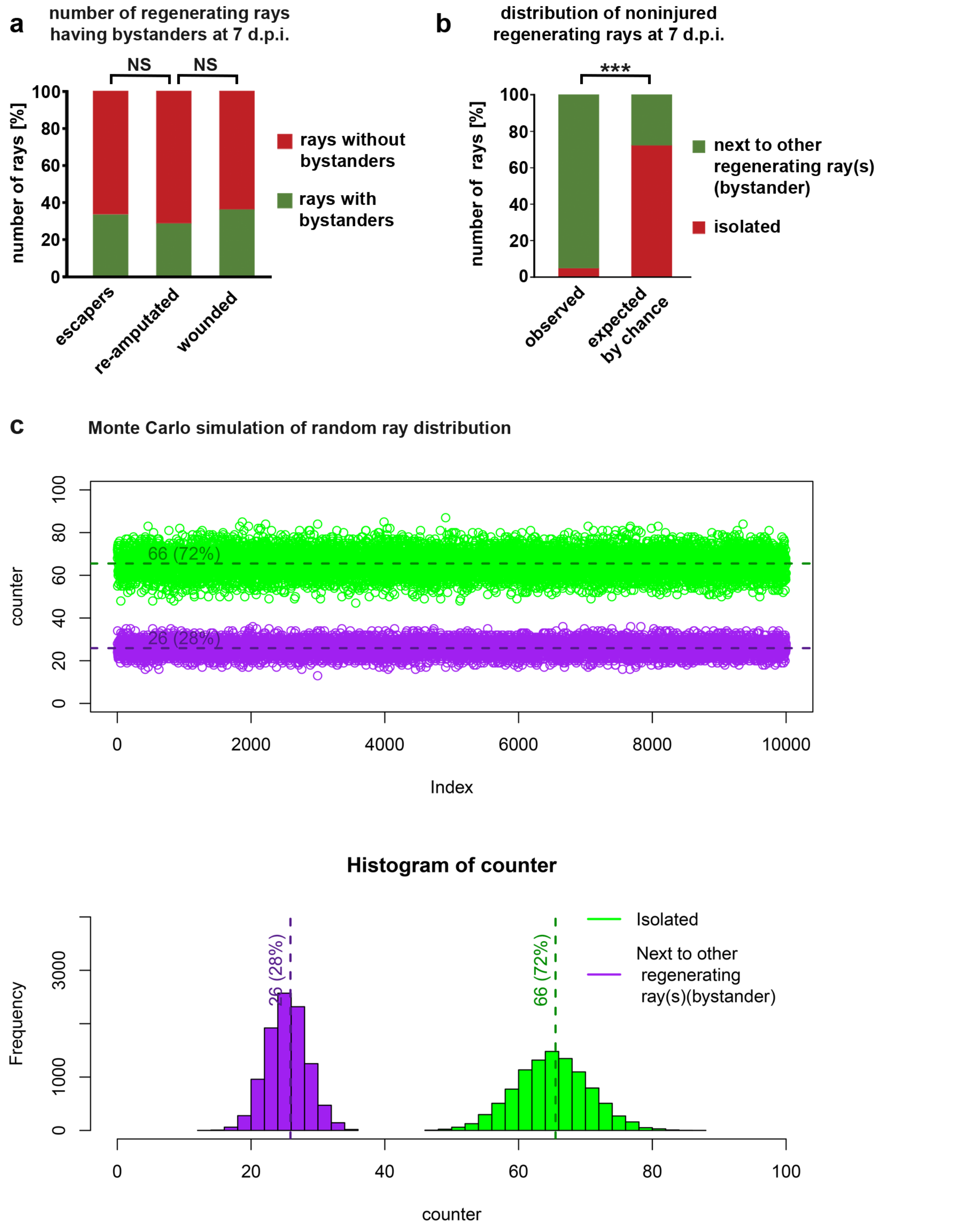

Supplementary Figure 9. Ray regeneration can induce bystander regeneration in adjacent rays

a Quantification of bystander growth in fin rays neighboring regenerating rays. *hsp70l*:dnFgfr1-EGFP fish were subjected to the experimental scheme shown in Figure 5d, and at 7 d.p.i. all regenerating rays were analyzed for the presence of additional, neighboring regenerating rays. This was done separately for rays that had escaped full blockage by dnFGFR1-GFP expression (rays that

showed signs of growth at 0 d.p.i.), and rays after re-amputation or epidermal wounding. The frequency of rays regenerating with or without such bystanders is plotted. n (escapers) = 69 rays, 61 fish; n (re-amputated) = 28 rays, 28 fish; n (wounded) = 50 rays, 48 fish. Note that frequencies are not significantly different between groups (Chi-Square test), indicating that bystander-growth was not due to inadvertent injury of rays neighboring those that we re-amputated or epidermally wounded. **b** Uninjured rays regenerate almost exclusively next to other regenerating rays, a finding that differs significantly from the distribution expected by chance (Monte Carlo simulation; p=0.0001). The frequency by which uninjured regenerating rays at 7 d.p.i. were found in isolation versus next to at least one regenerating ray that was classified as escaper, re-amputated, or epidermally wounded is plotted and compared with the distribution expected by chance as determined by Monte Carlo simulation. n (observed) = 165 rays, 45 fish. Thus, growth of bystanders did not occur by chance, but was induced by regeneration of their neighbors. **c** Monte Carlo simulation (10,000 iterations) assuming random distribution of regenerating ("1") and non-regenerating ("0") rays at frequencies observed in wounding experiments 1 & 2. Plotted is the frequency (counter) of rays being isolated ("010", green) or found in groups ("0110", "01110" and so forth, magenta). Histogram of counter shows that in 72% of cases, rays are expected to be regenerating in isolation if they were randomly distributed

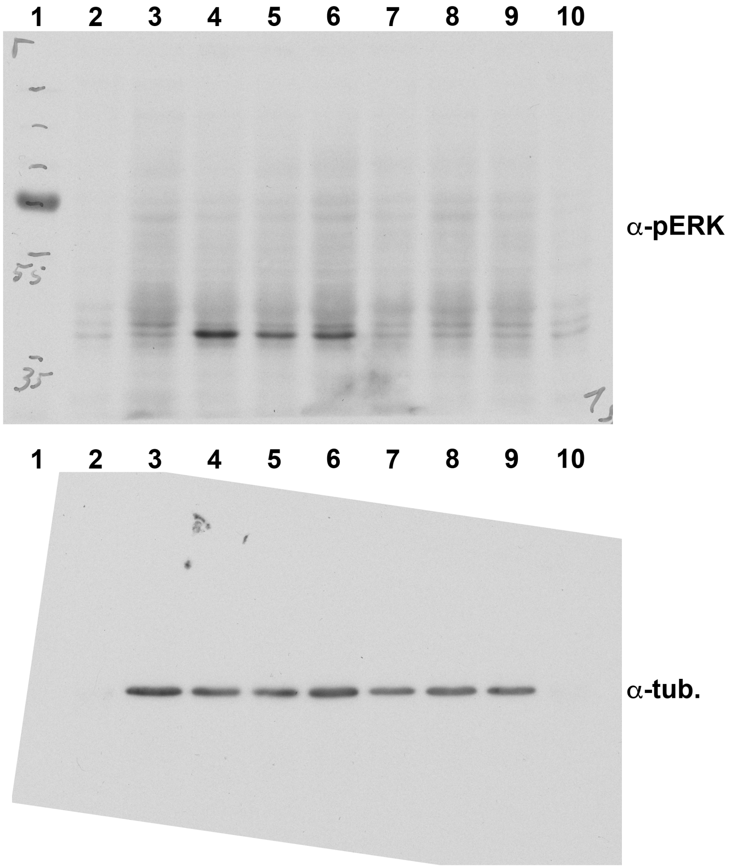

1: MW-Marker
2: Fill sample (under-loaded)
3: Intact control
4: 0.25 h.p.a.
5: 0.5 h.p.a.
6: 3 h.p.a.
7: 1 d.p.a.
8: 3 d.p.a.
9: 7 d.p.a.
10: Fill sample (under-loaded)

Supplementary Figure 10. Uncropped version of the western blot used in Figure 2a

pERK levels throughout the first week of regeneration. An antibody against α-tubulin (α-tub.) was used as a loading control

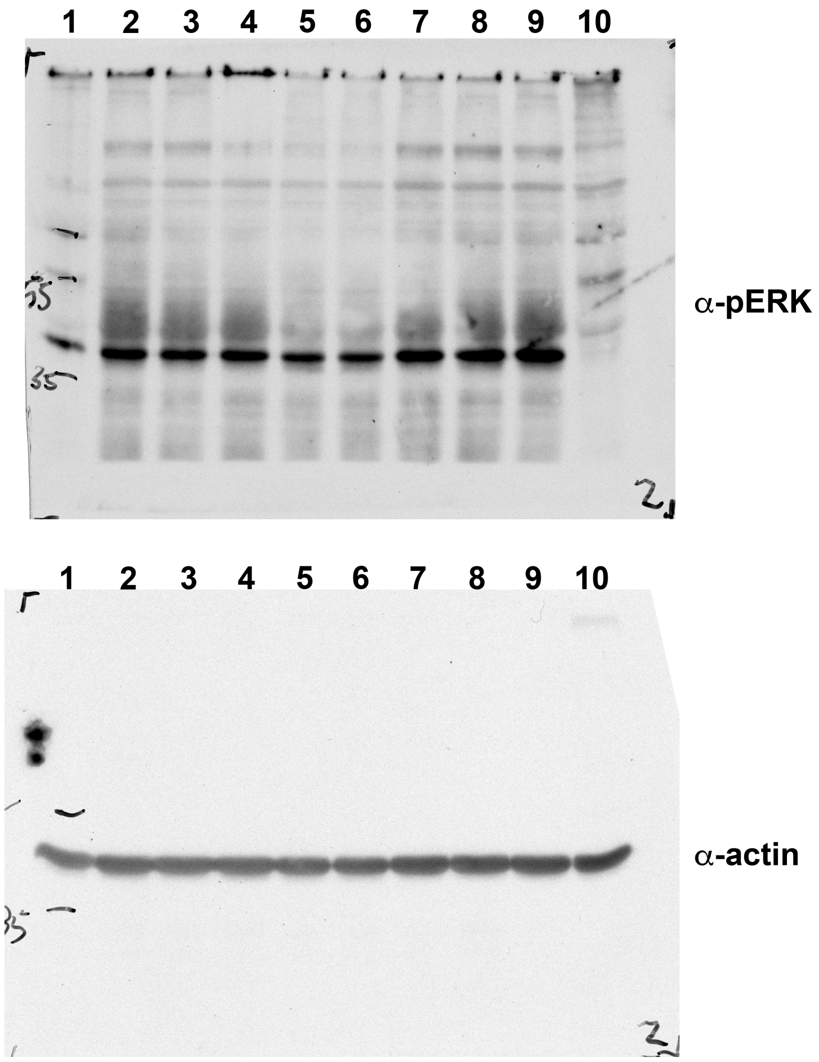

1: MW-marker+fill sample
2: unirradiated 3 h.p.a. Replicate 1
3: 1 d.p.irr. 3 h.p.a. Replicate 1
4: 3 d.p.irr. 3 h.p.a. Replicate 1
5: DMSO control 3 h.p.a.
6: Cycloheximide-treated 3 h.p.a.
7: unirradiated 3 h.p.a. Replicate 2
8: 1 d.p.irr. 3 h.p.a. Replicate 2
9: 3 d.p.irr. 3 h.p.a. Replicate 2
10: Fill sample

Supplementary Figure 11. Uncropped version of the western blot used in Figure 2b
pERK levels at 3 h.p.a. in irradiated and cycloheximide-treated animals as well as corresponding controls. An antibody against actin (α-actin) was used as a loading control; d.p.irr. days post irradiation

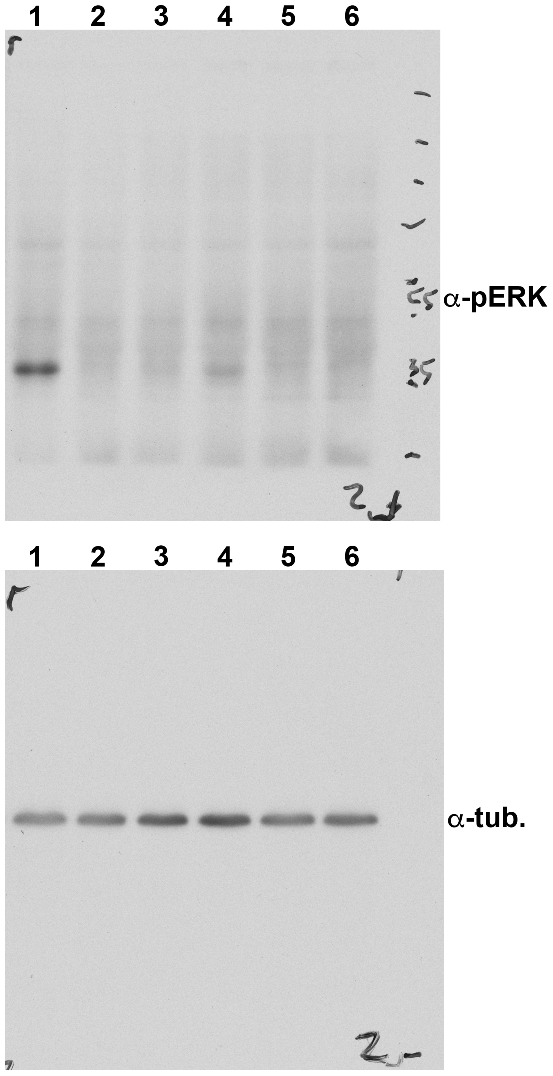

1: DMSO control 3 h.p.a.
2: PD 3 h.p.a.
3: U0 3 h.p.a.
4: DMSO control 3 d.p.a.
5: PD 3 d.p.a.
6: U0 3 d.p.a.

Supplementary Figure 12. Uncropped version of the western blot used in Figure 2d
pERK levels in DMSO and MEK inhibitor-treated animals at 3 h.p.a. and 3 d.p.a. An antibody against α-tubulin (α-tub.) was used as a loading control

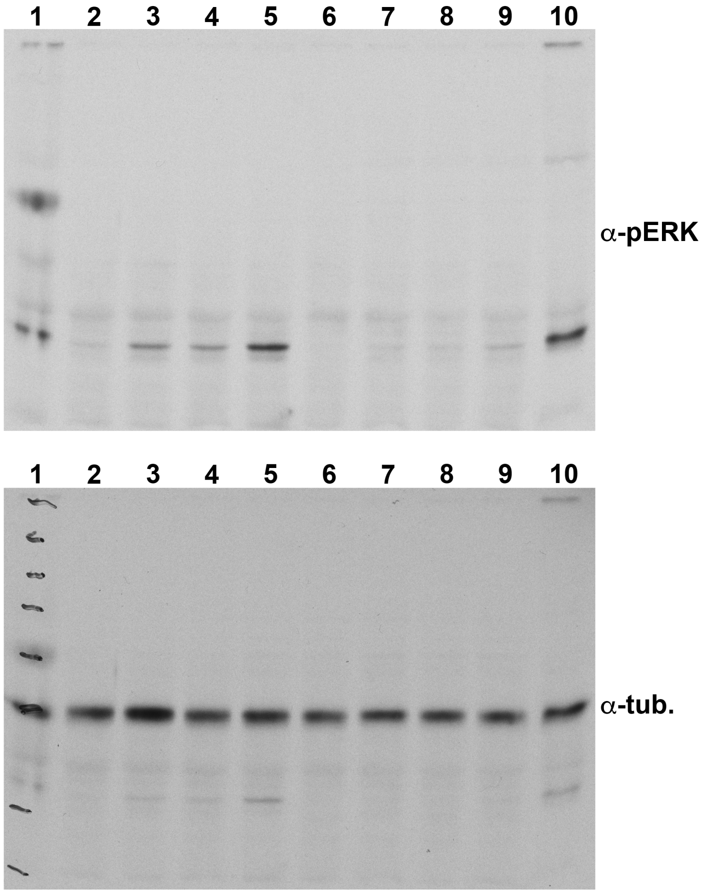

1: MW-Marker + fill sample
2: Dormant tail no re-wounding, DMSO
3: Dormant tail amputation, DMSO
4: Dormant tail single incision, DMSO
5: Dormant tail triple incision, DMSO
6: Dormant tail no re-wounding, PD
7: Dormant tail amputation, PD
8: Dormant tail single incision, PD
9: Dormant tail triple incision, PD
10: Fill sample

Supplementary Figure 13. Uncropped version of the western blot used in Figure 3h

pERK levels in “dormant” tails at 3 h.p.w. with DMSO or PD treatment and respective uninjured controls. An antibody against α-tubulin (α-tub.) was used as a loading control

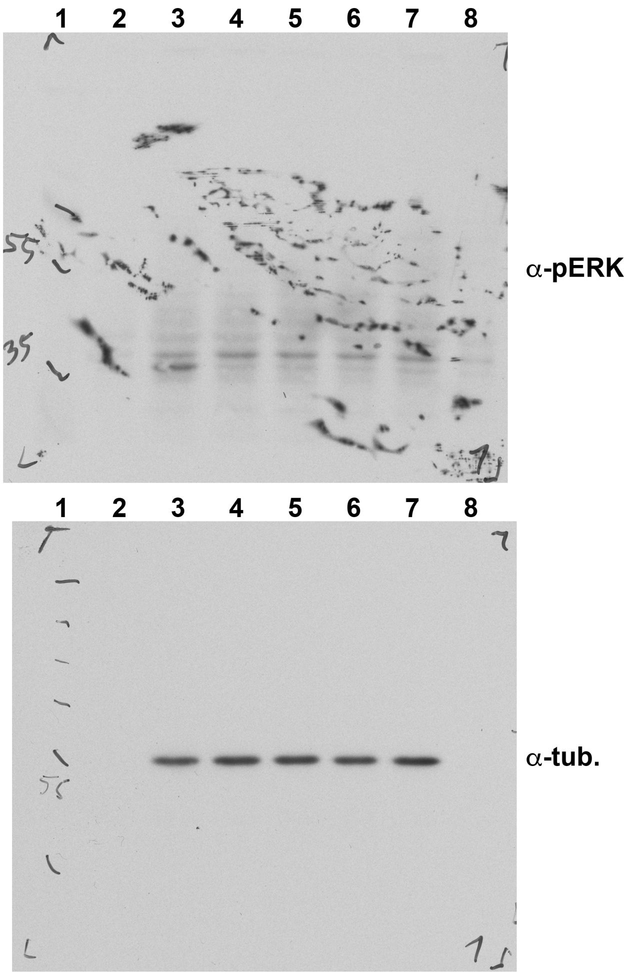

1: MW-Marker
2: Fill sample (under-loaded)
3: DMSO 1 day pre-treatment, 10 m.p.a.
4: PD 1 day pre-treatment, 10 m.p.a.
5: PD no pre-treatment, 10 m.p.a.
6: U0 1 day pre-treatment, 10 m.p.a.
7: U0 no pre-treatment, 10 m.p.a.
8: Fill sample (under-loaded)

Supplementary Figure 14. Uncropped version of the western blot used in Supplementary Figure 1a

pERK levels at 10 minutes post-amputation (m.p.a.), with or without 1 day pre-treatment with DMSO or a MEK inhibitor. An antibody against α-tubulin (α-tub.) was used as a loading control

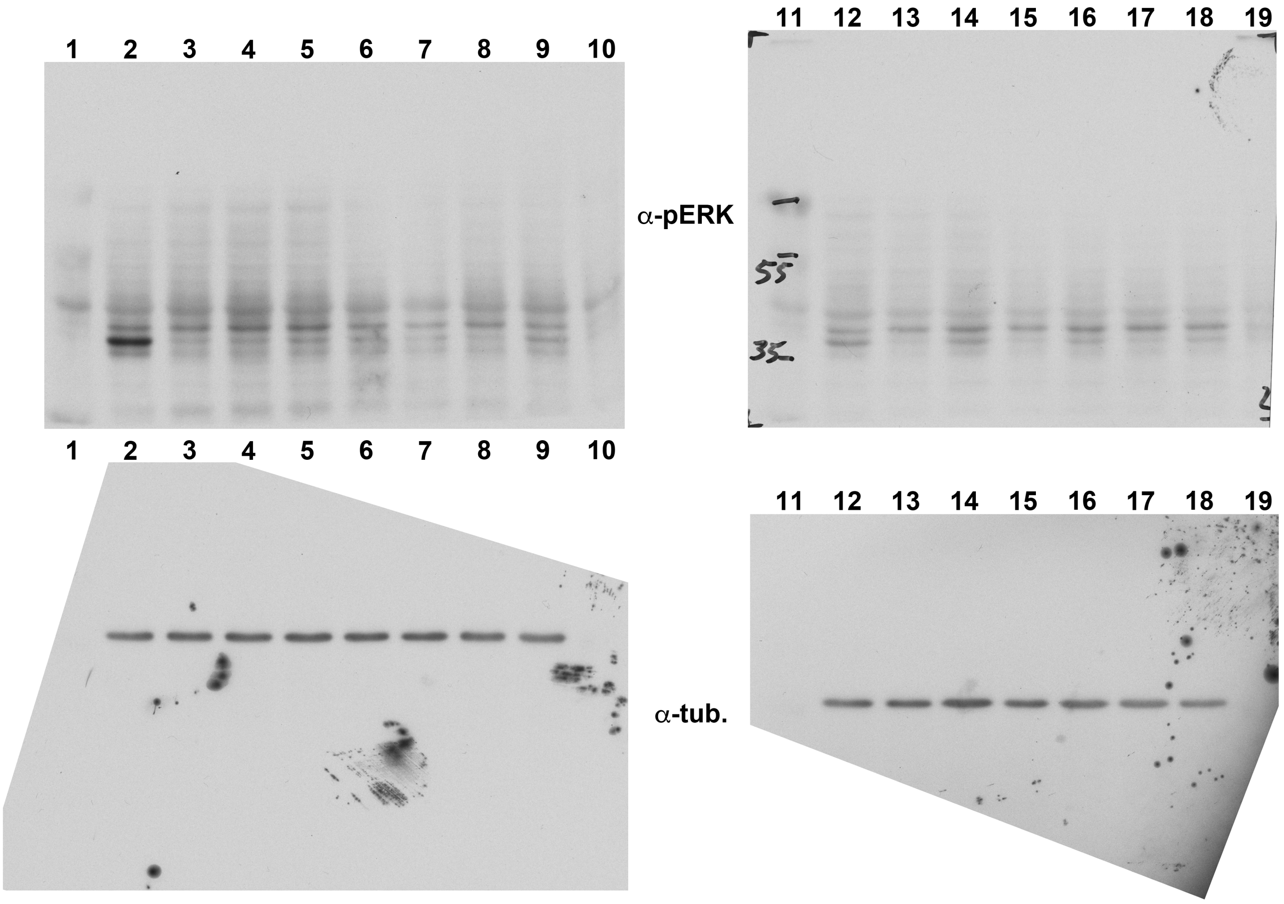

1: MW-Marker + fill sample
2: DMSO 3 h.p.a.
3: PD 3 h.p.a. continued
4: PD 3 h.p.a. washout
5: U0 3 h.p.a. continued
6: U0 3 h.p.a. washout
7-9: unrelated samples
10: Fill sample

11: MW-Marker + fill sample
12: DMSO 6 h.p.a.
13: PD 6 h.p.a. continued
14: PD 6 h.p.a. washout
15: U0 6 h.p.a. continued
16: U0 6 h.p.a. washout
17+18: unrelated sample
19: Fill sample

Supplementary Figure 15. Uncropped version of the western blot used in Supplementary Figure 1b

pERK levels at 3 and 6 h.p.a., with or without washout of MEK inhibitor, and corresponding controls. An antibody against α-tubulin (α-tub.) was used as a loading control

1 2 3 4 5 6 7 8 9

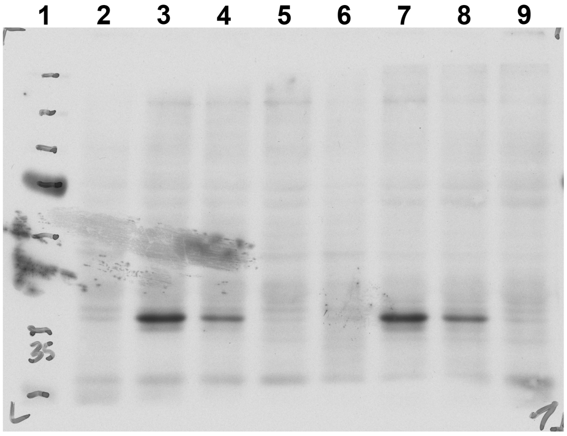

α-pERK

1 2 3 4 5 6 7 8 9

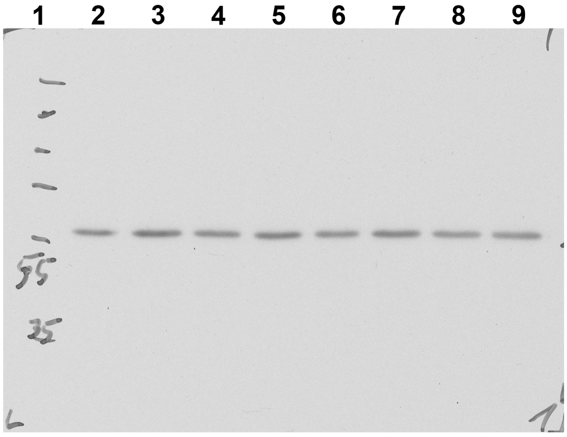

α-tub.

1: MW-Marker
2: 0 h.p.w. Replicate 1
3: 0.5 h.p.w. Replicate 1
4: 3 h.p.w. Replicate 1
5: 3 d.p.w. Replicate 1
6: 0 h.p.w. Replicate 2
7: 0.5 h.p.w. Replicate 2
8: 3 h.p.w. Replicate 2
9: 3 d.p.w. Replicate 2

Supplementary Figure 16. Uncropped version of the western blot used in Supplementary Figure 5a

pERK levels in DMSO and MEK inhibitor-treated animals at 0.5 hours, 3 hours and 3 days after incision. An antibody against α-tubulin (α-tub.) was used as a loading control

Supplementary References

1 Wenemoser, D. & Reddien, P. W. Planarian regeneration involves distinct stem cell responses to wounds and tissue absence. *Dev Biol* **344**, 979-991, doi:S0012-1606(10)00837-7 [pii] 10.1016/j.ydbio.2010.06.017 (2010).

2 Gavino, M. A., Wenemoser, D., Wang, I. E. & Reddien, P. W. Tissue absence initiates regeneration through Follistatin-mediated inhibition of Activin signaling. *Elife* **2**, e00247, doi:10.7554/eLife.00247 00247 [pii] (2013).

3 Zayas, R. M., Cebria, F., Guo, T., Feng, J. & Newmark, P. A. The use of lectins as markers for differentiated secretory cells in planarians. *Dev Dyn* **239**, 2888-2897, doi:10.1002/dvdy.22427 (2010).

4 Wurtzel, O. *et al.* A Generic and Cell-Type-Specific Wound Response Precedes Regeneration in Planarians. *Developmental cell* **35**, 632-645, doi:10.1016/j.devcel.2015.11.004 (2015).

Supplementary Methods

Unless otherwise indicated, the numbers of animals used are indicated on the figures.

<u>Figure 2:</u>

a) 5 worms (15 fragments) per condition, 3 independent experiments

b) 5 worms (15 fragments) per condition, 2 independent experiments

d) 5 worms (15 fragments) per condition, 2 independent experiments

e) minimum 4 independent experiments

f-g) 1 experiment

h) phospho-Histone-3 stains, 3 independent experiments, quantified in Supplementary Figure 3d; SMEDWI-1 stains, 3 independent experiments

<u>Figure 3:</u>

b) 1 experiment

c) *notum* expression, 1 experiment; live animals, 7 independent experiments

d) uninjured+PD, 4 independent experiments, injured+PD, 5 independent experiments

e) quantification of animals shown in Fig. 3c and Supplementary Fig. 6

f) 1 experiment

g) feeding and locomotion assays, 2 independent experiments at 13 d.p.w.

h) 6-10 fragments per condition, 2 independent experiments

<u>Figure 4:</u>

a) 4 independent experiments

b) *sFRP-1* and *ndk* expression, 1 experiment

c) 3 independent experiments

d) 3 independent experiments

e) 2 independent experiments

Supplementary Figure 1:

a) 5 worms (15 fragments) per condition, 1 experiment

b) 5 worms (15 fragments) per condition, 1 experiment

c) live animals, minimum 5 independent experiments; *prcn* expression, 3 independent experiments; *PC2* expression, 2 independent experiments; *sFRP-1* expression, 1 experiment for head and trunks, 2 independent experiments for tails

Supplementary Figure 2:

Minimum 2 independent experiments for scoring live animals as well as *sFRP-1* and *notum* expression

Supplementary Figure 3:

a) 1 experiment

b) analysis based on 3 biological replicates, each consisting of minimum 5 animals (15 fragments) and 2-3 technical replicates.

c-d) quantification from 3 independent experiments, please refer to Figure 4c and 2h for representative images of phosphoHistone-3 stains in head and tail fragments, respectively

e) 3 independent experiments

f) Results from two independent experiments were pooled

Supplementary Figure 4:

a) 2 independent experiments

b) 2 independent experiments

Supplementary Figure 5:

a) 5 worms per condition, 2 independent experiments

b) 2 independent experiments, numbers refer to injuries (2 injuries per animal)

c) 1 experiment, numbers refer to injuries (2 injuries per animal)

d) analysis based on 3 biological replicates, each consisting of minimum 5 animals (15 fragments) and 2 technical replicates.

e) 3 independent experiments

f) 1 experiment

g) 2 independent experiments, numbers refer to injuries (2 injuries per animal)

h) quantification from 3 independent experiments

Supplementary Figure 6:

a) live animals, 7 independent experiments; SMEDWI-1 staining, phosphoHistone-3 staining, 1 experiment; TUNEL staining, 2 independent experiments

b) live animals: uninjured+PD, 4 independent experiments, injured+PD, 5 independent experiments; pH3, TUNEL, 1 experiment

c) *sFRP-1* and *PC2* expression, 1 experiment; *MAT* expression, 2 independent experiments

Supplementary Figure 7:

1 experiment
